# Laevofolinic acid, 5-fluorouracil, cyclophosphamide and escalating doses of epirubicin with granulocyte colony-stimulating factor support in locally advanced and/or metastatic breast carcinoma: a phase I-II study of the Southern Italy Oncology Group (GOIM).

**DOI:** 10.1038/bjc.1995.494

**Published:** 1995-11

**Authors:** G. Colucci, S. Romito, V. Gebbia, G. Pacilio, F. Giotta, A. Testa, G. Pezzella, E. Durini, B. Agostara, S. Cariello

**Affiliations:** Oncological Institute, Bari, Italy.

## Abstract

Sixty-four consecutive patients with locally advanced (n = 7) or metastatic breast cancer (n = 57), were treated with a combination of laevofolinic acid 100 mg m-2 plus 5-fluorouracil 340 mg m-2 i.v. on days 1-3, cyclophosphamide 600 mg m-2 i.v. on day 1 and epirubicin 90 mg m-2 i.v. on day 1. Epirubicin dose was progressively escalated by 10 mg m-2 per cycle up to 120 mg m-2 in the absence of dose-limiting toxicities. Granulocyte colony-stimulating factor (G-CSF) was given subcutaneously in order to prevent neutropenia. Epirubicin dosage could be increased to 100 mg m-2 in 53 patients (87%), to 110 mg m-2 in 31 patients (51%) and to 120 mg m-2 in 18 cases (30%). In most patients the dose-limiting toxicity was represented by myelosuppression. A statistically significant correlation was found between median white blood count (WBC) or absolute neutrophil count (ANC) nadir and epirubicin dose level (P = 0.009; P = 0.008). Moreover, a statistically significant correlation was observed between the number of chemotherapeutic cycles, nadir ANC and WBC and the occurrence of anaemia and thrombocytopenia of increasing severity. These data suggest the occurrence of progressive cumulative bone marrow toxicity. Although patients who reached different epirubicin levels showed differences in mean dose intensity, such differences were not statistically significant. No correlation was found between the increase in dose intensity and type, rate or duration of objective responses. In patients with metastatic breast cancer the overall response rate was 72% (95% CL 66-78%) with a 25% complete response rate. Median duration of response was 10 and 13 months respectively for complete and partial responses. All patients with locally advanced breast cancer had an objective response and underwent radical mastectomy. Projected median survival of the whole series of patients with metastatic breast cancer was 20 + months. These data demonstrate that the combination of 5-fluorouracil with laevofolinic acid, cyclophosphamide and epirubicin is very active against metastatic breast cancer. Use of G-CSF allows epirubicin dosage to be increased up to 120 mg m-2 cycle-1, but its use may be linked to the occurrence of sometimes severe cumulative haematological toxicity.


British Journal of Cancer (1995) 72, 1245-1250

© 1995 Stockton Press All rights reserved 0007-0920/95 $12.00

### Laevofolinic acid, 5-fluorouracil, cyclophosphamide and escalating doses of epirubicin with granulocyte colony-stimulating factor support in locally advanced and/or metastatic breast carcinoma: a phase I-II study of the Southern Italy Oncology Group (GOIM)

G Colucci[1], S Romito[1], V Gebbia[2], G Pacilio[3], F Giotta[1], A Testa[2], G Pezzella[4], E Durini[5], B Agostara[6], S Cariello[7], S Palazzo[8] and N Gebbia[2]

[1]*Oncological Institute, Bari;* [2]*Service of Chemotherapy, University of Palermo;* [3]*Division of Oncology, Caldarelli Hospital, Napoli;* [4]*Service of Oncology, 'SS Annunziata' Hospital, Taranto;* [5]*Service of Oncology, 'Panico' Hospital, Tricase;* [6]*Service of Chemotherapy, 'Oncological Hospital', Palermo;* [7]*Division of Oncology, 'S Leonardo' Hospital, Salerno;* [8]*Service of Oncology, 'Dell'Annunziata' Hospital, Cosenza, Italy*

**Summary** Sixty-four consecutive patients with locally advanced ($n = 7$) or metastatic breast cancer ($n = 57$), were treated with a combination of laevofolinic acid 100 mg m$^{-2}$ plus 5-fluorouracil 340 mg m$^{-2}$ i.v. on days 1-3, cyclophosphamide 600 mg m$^{-2}$ i.v. on day 1 and epirubicin 90 mg m$^{-2}$ i.v. on day 1. Epirubicin dose was progressively escalated by 10 mg m$^{-2}$ per cycle up to 120 mg m$^{-2}$ in the absence of dose-limiting toxicities. Granulocyte colony-stimulating factor (G-CSF) was given subcutaneously in order to prevent neutropenia. Epirubicin dosage could be increased to 100 mg m$^{-2}$ in 53 patients (87%), to 110 mg m$^{-2}$ in 31 patients (51%) and to 120 mg m$^{-2}$ in 18 cases (30%). In most patients the dose-limiting toxicity was represented by myelosuppression. A statistically significant correlation was found between median white blood count (WBC) or absolute neutrophil count (ANC) nadir and epirubicin dose level ($P = 0.009$; $P = 0.008$). Moreover, a statistically significant correlation was observed between the number of chemotherapeutic cycles, nadir ANC and WBC and the occurrence of anaemia and thrombocytopenia of increasing severity. These data suggest the occurrence of progressive cumulative bone marrow toxicity. Although patients who reached different epirubicin levels showed differences in mean dose intensity, such differences were not statistically significant. No correlation was found between the increase in dose intensity and type, rate or duration of objective responses. In patients with metastatic breast cancer the overall response rate was 72% (95% CL 66-78%) with a 25% complete response rate. Median duration of response was 10 and 13 months respectively for complete and partial responses. All patients with locally advanced breast cancer had an objective response and underwent radical mastectomy. Projected median survival of the whole series of patients with metastatic breast cancer was 20 + months. These data demonstrate that the combination of 5-fluorouracil with laevofolinic acid, cyclophosphamide and epirubicin is very active against metastatic breast cancer. Use of G-CSF allows epirubicin dosage to be increased up to 120 mg m$^{-2}$ cycle$^{-1}$, but its use may be linked to the occurrence of sometimes severe cumulative haematological toxicity.

**Keywords**: granulocyte colony-stimulating factor; epirubicin; fluorouracil; cyclophosphamide; metastatic breast cancer

Despite considerable progress achieved in the last two decades in clinical oncology, metastatic breast carcinoma (MBC) still remains a fatal illness. Results of palliative polychemotherapy for MBC are still considered largely unsatisfactory (Henderson, 1987), but their translation to an adjuvant setting has led to important advances in the treatment of earlier stages (Henderson *et al*., 1988).

Doxorubicin is commonly considered as the most active chemotherapeutic drug for the treatment of MBC, being able to induce a nearly 40% overall response rate when employed as a single agent (Hoogstraten, 1975). The doxorubicin 4'-epimer epirubicin (EPI) has a more favourable therapeutic index than doxorubicin, since at equimolar doses it shows identical clinical activity with less haematological and cardiac toxicity (French Epirubicin Study Group, 1988; Bonadonna *et al*., 1993).

Several data from preclinical studies have shown that anthracycline drugs display a steep dose-response curve (Razak *et al*., 1972; Frei and Cannellos, 1980). Clinical studies on acute myeloid leukaemia and bone sarcomas have shown that higher doses of anthracyclines are associated with higher complete response rates and improved survival (Cortes *et al*., 1978; Yates *et al*., 1982). Moreover, retrospective analyses of clinical trials employing the cyclophosphamide, methotrexate and 5-fluorouracil (CMF) or 5-fluorouracil, doxorubicin and cyclophosphamide (FAC) regimens in MBC carried out independently by Hryniuk (1987) and Tannock *et al*. (1988) showed that an increased dose intensity is associated with a better response rate and an increased quality of life. These data have been partially confirmed, at least in terms of response rates, in several clinical investigations on MBC treated with EPI (Hortobagyi, 1990; French Epirubicin Study Group, 1991; Habeshaw *et al*., 1991; Marschner *et al*., 1994). For these reasons new regimens containing high-dose EPI have been developed with the aim of improving clinical results with acceptable toxicity (Hortobagyi, 1990; Ferguson *et al*., 1993; Marschner *et al*., 1994).

The combination of 5-fluorouracil (5-FU), cyclophosphamide (CTX) and EPI employed in a wide dose range, the so-called' FEC regimen, has been largely employed in the treatment of MBC (French Epirubicin Study Group, 1988, 1991; Italian Multicenter Breast Study with Epirubicin, 1988). Hortobagyi (1990) recently reported substantial differences in response rates with the FEC regimen depending on programmed EPI dosage: the FEC regimen with EPI at a dosage of 100 mg m$^{-2}$ cycle$^{-1}$ obtained a 79% overall response rate as compared with the 46% rate achieved with the same regimen with EPI at 50 mg m$^{-2}$ cycle$^{-1}$.

The therapeutic activity of 5-FU against gastrointestinal malignancies may be significantly enhanced by increasing the intracellular pool of laevofolates (*l*-FA), which is clinically achieved by administering variable amounts of exogenous

Correspondence: V Gebbia, Service of Chemotherapy, Institute of Pharmacology, Policlinico, via del Vespro n 129, Palermo 90127, Italy
Received 3 April 1995; revised 12 June 1995; accepted 22 June 1995

l-FA to cancer patients (Allegra, 1991). The combination of l-FA and 5-FU has also been tested in MBC patients, achieving response rates ranging from 17% to 48%, largely depending on the extent of pretreatment (Loprinzi, 1989). With the aim of improving clinical results some authors have tested a modification of the FAC or FEC regimens employing 5-FU modulated by l-FA with interesting results (Zaniboni *et al.*, 1989; Palmeri *et al.*, 1991; Parnes *et al.*, 1991; Gebbia *et al.*, 1992).

It has recently been reported that human recombinant granulocyte colony-stimulating factor (G-CSF) used subcutaneously is able to reduce the duration of severe leucopenia and the incidence of mucositis and febrile events commonly associated with intensive chemotherapy (Bronchud *et al.*, 1989; Gebbia *et al.*, 1993). G-CSF allowed a timely readministration or even anticipation of subsequent cycles obtained an increase in dose intensity.

According to the above-reported rationale, we carried out a multicentre study with the combination of CTX, 5-FU/l-FA, and escalating doses of EPI with G-CSF support with the aims of increasing anthracycline dosage to a level at which dose-limiting toxicity could be identified, shortening the inter-cycle interval, and improving dose intensity, as well as determining the clinical efficacy and toxicity of such a regimen.

### Patients and methods

### *Eligibility criteria*

Since the aims of this study included both the identification of dose-limiting toxicity and the evaluation of clinical efficacy in terms of objective regression rate, response duration and patient survival, measurable disease out with radiotherapy fields according to the WHO criteria (Miller *et al.*, 1981) was a necessary elegibility criterion. Before entry into the study all patients also had to fulfil the following eligibility criteria: written informed consent as required by the ethical committee of our institutions; histological diagnosis of advanced breast carcinoma; age > 18 and $\leqslant$65 years; Karnofsky performance status $\geqslant$60; life expectancy $\geqslant$3 months; adequate bone marrow function [WBC $\geqslant$4000 cells $\mu l^{-1}$, platelets (PTL) > 120 000 cells $\mu l^{-1}$, haemoglobin (Hb) > 10 g $dl^{-1}$; serum bilirubin $\leqslant$1.2 mg $dl^{-1}$; serum transaminases < twice the normal value; serum creatinine $\leqslant$1.2 mg $dl^{-1}$; blood urea nitrogen (BUN) $\leqslant$50 mg $dl^{-1}$]; normal cardiac function as evaluated by ECG and echocardiography; no signs or symptoms of brain metastases; absence of severe uncontrolled cardiovascular, metabolic, neurological, respiratory, liver or renal disease; absence of a second malignancy except for *in situ* carcinoma of the cervix or cutaneous basalioma; geographical accessibility in order to guarantee a correct follow-up.

Previous adjuvant chemotherapy without anthracycline or anthrachinonic drugs was permitted, but chemotherapeutic treatment had to be withdrawn at least 12 months before entry into the study. Previous hormonal therapies were also permitted but they had to be discontinued at least 4 weeks before entry.

### *Staging*

Pretreatment staging procedures included: history and physical examination, haemocromocytometric parameters, routine serum chemistry tests, ECG, echocardiography and/or radionuclide gated scan with evaluation of the left ventricular ejection fraction (LVEF), chest radiograph, abdominal sonograms, and technetium-99 bone scan. Computerised tomography (CT) scan of the involved areas was also required for a better definition of disease extension and evaluation of objective response. CT scan was performed before chemotherapy, after the first three cycles, and then every two or three cycles in responding patients. ECG and evaluation of LVEF were performed every cycle and every other cycle respectively to monitor cardiac function closely.

### *Treatment plan and toxicity*

The treatment schedule was: l-FA 100 mg $m^{-2}$ as intravenous bolus plus 5-FU 340 mg $m^{-2}$ i.v. over 15 min on days 1->3; CTX 600 mg $m^{-2}$ as intravenous bolus on day 1; and EPI 90 mg $m^{-2}$ i.v. bolus on day 1. G-CSF 5 $\mu g$ $kg^{-1}$ $day^{-1}$ was given subcutaneously for 12 days starting from day 5 of each cycle.

Toxicity was recorded according to the WHO criteria (Miller *et al.*, 1981). In each patient EPI dosage was tentatively escalated by 10 mg $m^{-2}$ $cycle^{-1}$ until unacceptable dose-limiting toxicity (DLT) ensued or the maximal dose of 120 mg $m^{-2}$ was reached. Interim blood cell counts were performed starting from day 12 of each cycle in order to increase EPI dosage by one dose level in the subsequent cycle. Blood cell counts were repeated every 2 days to establish the duration of myelosuppression.

The dose-limiting toxicity was represented by any of the following side-effects: nadir WBC < 1000 cells $\mu l^{-1}$ with ANC < 500 cells $\mu l^{-1}$ (WHO grade 4) for $\geqslant$5 days, PTL < 75 000 cells $\mu l^{-1}$ (> grade 2), precycle anaemia > grade 2, mucositis or other extrahaematological toxicity $\geqslant$grade 3, or toxicity-related delay > 8 days. If one or more of the above mentioned side-effects occurred at any dose level, EPI dosage was not further increased and reduced by one dose level in the subsequent cycle.

As soon as the WBC and PTL reached acceptable values after the nadir the subsequent cycle of chemotherapy was tentatively given before day 21 with the aim of shortening the inter-cycle interval. If at planned recycling (day 21) persistence of mucositis, leucopenia (WBC < 4000 cells $\mu l^{-1}$) and/or thrombocytopenia (PTL $\leqslant$120 000 cells $\mu l^{-1}$) treatment was delayed until day 28. With the exception of alopecia and leucopenia, the occurrence of grade 4 toxicity of any kind led to patients' withdrawal from chemotherapy. Treatment was stopped if LVEF decreased by > 15% from basal level and/or patients developed initial signs of cardiac failure. Once EPI level was defined, the dose level was maintained for the remainder of the trial.

The first response evaluation was performed after the third cycle: in the case of complete objective tumour regression or stabilisation patients received a total of six cycles; in the case of progressive disease patients were withdrawn and followed up. In the case of partial response patients were treated for up to eight cycles and then followed up.

### *Responses and toxicity*

Patients were evaluated for objective response after three cycles according to the WHO response criteria (Miller *et al.*, 1981). Briefly, complete response (CR) was defined as the complete disappearance of all signs and symptoms of tumour for a minimum of 4 weeks; partial response (PR) was defined as a > 50% reduction in the sum of the largest perpendicular diameters of all measurable lesions for at least 4 weeks without a simultaneous increase in the size of any lesion or the appearance of any new metastases; stable disease (SD) as a $\leqslant$50% decrease or $\leqslant$25% increase in the size of tumoral deposits; and progressive disease (PD) as a > 25% increase in the size of tumour lesions and/or the appearance of any new metastases. For bone lesions a CR was defined as the complete disappearance of all lesions on radiography or scan for at least 4 weeks; PR was defined as a partial decrease in the size of lytic lesions, recalcification of lytic lesions or decreased density of blastic lesions (Miller *et al.*, 1981). Duration of objective responses was calculated starting from the first day of treatment until progressive disease was recorded. Survival was calculated from the first day of chemotherapy until death or was censored on the date of the last follow-up.

### Statistics

At entry all patients were communicated to the co-ordinating centre and all data were centrally monitored at the Oncological Institute of Bari. Objective tumour regressions were reported as relative rates with 95% confidence limits (95% CL). Univariate analysis of survival data according to the product limit (Kaplan-Meier) estimate was carried out. Comparison in survival distribution was made using the log-rank test. Calculation of dose intensity was carried out according to Hryniuk (1987) and the Wilcoxon rank sum test was used to evaluate the differences in EPI dose intensity.

### Results

### Patient population

The main clinical and demographic characteristics of enrolled patients are depicted in Table I. Briefly, between April 1992 and September 1993 64 consecutive patients with metastatic breast carcinoma (MBC, $n = 57$) or locally advanced inoperable breast cancer (LABC, $n = 7$) entered this study. Median age was 50 years, and median performance status according to the Karnofsky index was 80. Twenty-eight patients were premenopausal (44%), and 36 (56%) post-menopausal. Hormonal receptors were positive in 17 patients, negative in 16 and unknown in 31 cases. There were 45 ductal infiltrating carcinomas, ten lobular infiltrating carcinomas, and nine other histological types. Among patients with MBC the median disease-free interval from surgical operation to first recurrence was 3.2 years (range 1.2-5.4).

Among the 57 patients with MBC, visceral metastases represented the dominant site of disease in 32 patients (56%) and 45 patients (89%) presented more than one site of disease. Sites of disease included: nodes (40 patients), bone (23 patients), breast (15 patients), liver (16 patients), lung (13 patients), skin (five patients), pleura (four patients), and pleural effusion (six patients). Pretreatments included: surgery in all cases; adjuvant chemotherapy without anthracyclines in 23 patients (40%); and hormonotherapy in 19 (33%). No patient had received chemotherapy for metastatic disease.

**Table I** Patient characteristics

| | |
|---|---|
| All enrolled patients | |
| Number | 64 (100%) |
| Age median (range) | 50 (27/65) |
| Performance status median (range) | 80 (60/100) |
| Menopausal status | |
| Premenopausal | 28 (44%) |
| Post-menopausal | 36 (56%) |
| Hormonal receptors | |
| Positive | 17 (27%) |
| Negative | 16 (25%) |
| Unknown | 31 (48%) |
| Patients with locally advanced breast carcinoma | 7 |
| Locally advanced | 4 |
| Inflammatory carcinoma | 3 |
| Patients with metastatic breast carcinoma | 57 (100%) |
| Pretreatments | |
| Surgery | 57 (100%) |
| Hormonotherapy | 19 (33%) |
| Radiotherapy | 13 (17%) |
| Chemotherapy (adjuvant) | 23 (40%) |
| Dominant site of disease | |
| Viscera | 32 (56%) |
| Bone | 15 (26%) |
| Soft tissue | 10 (17%) |
| Number of involved sites | |
| Single | 12 (21%) |
| Multiple | 45 (89%) |
| Two sites | 24 |
| Three sites | 14 |
| Four sites | 7 |

### Dose escalation and toxicity

Sixty-one patients were evaluable for toxicity since three patients were lost to follow-up soon after the first cycle. Overall, a total of 384 cycles (6.3 cycles per patient) were administered. No chemotherapy-related deaths were recorded.

As shown in Table II, it was possible to increase EPI dosage from the starting dose of 90 mg $m^{-2}$ given at the first cycle to the first step of 100 mg $m^{-2}$ in 53 patients (87%). EPI dosage could not be increased because of prolonged grade 4 leucopenia with neutropenia in four patients, and because of $>$grade 2 thrombocytopenia in four other patients. Subsequently, EPI dosage was further increased to 110 mg $m^{-2}$ in 31 patients (51%), while it was left unchanged (100 mg $m^{-2}$) or reduced (90 mg $m^{-2}$) in 22 patients because of the occurrrence of grade 4 leucopenia, $>$grade 2 thrombocytopenia, $\geqslant$grade 2 anaemia, grade 3 stomatitis or a combination of these toxicities. Eighteen patients (30%) reached the final step of 120 mg $m^{-2}$. In 13 patients EPI was not increased to 120 mg $m^{-2}$ because of grade 4 leucopenia (three patients), thrombocytopenia $>$grade 2 (three patients), or prolonged grade 3 stomatitis (two patients). Dose escalation of EPI up to 120 mg $m^{-2}$ was also not achievable in four patients who dropped out of the study because of progressive cancer. The differences between median WBC and ANC counts recorded at the four different levels of EPI were statistically significant ($P = 0.006$; $P = 0.005$ respectively). Moreover, a clear correlation was found between EPI dose

**Table II** Toxicity according to EPI dosage

| *EPI level (mg $m^{-2}$)* | *No. of patients* | *Median number of cells $mm^{-3}$ at nadir* WBC | ANC | PTL (n × 10) | *Median Hb g%*[a] | *Dose-limiting toxicity*[b] *Cause* | *No. of patients affected* |
|---|---|---|---|---|---|---|---|
| 90 | 61 (100%) | 2557 | 1240 | 160 | 11.5 | Leucopenia | 4 |
| | | | | | | PTL | 4 |
| 100 | 53 (87%) | 2303 | 900 | 125 | 10.4 | Leucopenia | 12 |
| | | | | | | PTL | 4 |
| | | | | | | Anaemia | 5 |
| | | | | | | Mucositis | 1 |
| 110 | 31 (51%) | 1840 | 750 | 106 | 9.7 | Leucopenia | 5 |
| | | | | | | PTL | 2 |
| | | | | | | Mucositis | 2[c] |
| 120 | 18 (30%) | 1350 | 650 | 84 | 9.0 | Leucopenia | 14 |
| | | | | | | PTL | 4 |
| | | | | | | Anaemia | 3 |

[a]Hb values reported in the Table are not those recorded at nadir but median values recorded just before the next cycle of chemotherapy. [b]These data refer to those patients in whom EPI dosage could not be increased because of unacceptable toxicity; the highest grade toxicity per patient was reported as dose-limiting toxicity. [c]Escalation of EPI dosage to 120 mg $m^{-2}$ was not achievable in a further four patients because they were off-study owing to progressive disease.

level and median nadir counts (WBC: $r = -0.991$, $P = 0.009$; ANC $r = -0.961$, $P < 0.008$). Fourteen patients required hospitalisation and i.v. antibiotics during treatment due to severe febrile leucopenia.

Myelosuppression was by far the most frequent side-effect since all patients experienced leucopenia and/or thrombocytopenia and/or anaemia of some grade. Moreover, myelosuppression represented the DLT of EPI in combination with CTX and 5-FU/*l*-FA in the vast majority of patients with the exception of three patients who showed severe extrahaematological side-effects (stomatitis). Anaemia, as recorded before any cycle of chemotherapy, was recorded in 46 patients (75%), six of whom had grade 3 (10%) and two (4%) grade 4. Thrombocytopenia was observed in 34 patients (58%), of whom 13 had grade 3 and three grade 4. Overall, nine patients required RBC transfusion due to anaemia, and three platelet transfusion.

Figure 1 shows the median values of WBC and ANC before starting chemotherapy, at nadir and before any cycle. No correlation was found between the median WBC and ANC counts at nadir and the number of chemotherapeutic cycles. On the other hand, a statistically significant correlation was found between the number of chemotherapy cycles and median values of WBC ($r = -0.824$, $P = 0.012$) or ANC ($r = 0.842$, $P = 0.009$) recorded before any cycle of chemotherapy. No statistical difference was found between median nadir WBC or ANC values recorded at any cycle. On the other hand, the WBC curve showed a significant ($P = 0.002$) decrease in median WBC levels after cycle 5 which may suggest the occurrence of cumulative toxicity and/or a mild reduction of marrow protection despite the use of G-CSF.

Figure 2 shows PTL and Hb values plotted against number of cycles of chemotherapy. Analysis of Hb levels shows a remarkable progressive toxicity with a median reduction of Hb value of 2.7 g $dl^{-1}$ between pretreatment and final values ($P < 0.005$). A significant statistical correlation was found between number of cycles and development of anaemia ($r = -0.938$, $P = 0.0001$). Such an effect was less evident for PTL values which showed decrease at nadir with a rapid return to starting values and no statistically significant differences between pretreatment and final values. However, analysis of nadir PTL values showed a statistically significant correlation between thrombocytopenia and the number of chemotherapeutic cycles ($r = -0.709$, $P = 0.049$). Increase in the frequency of cycle administration was possible in 20 patients which showed shortening of the programmed inter-cycle interval to a median of 18 days (range 16–20). In most cases this was possible during the first three cycles after which cumulative bone marrow toxicity hampered shortening of the inter-cycle interval.

The median cumulative dosage of EPI was 645 mg $m^{-2}$ per patient with a median dose intensity of 38.41 mg $m^{-2}$ $week^{-1}$ which represented a 28% increase over the starting dosage. In the group of patients who could not receive EPI over 90 mg $m^{-2}$ the mean dose intensity was 33.38 mg $m^{-2}$ $week^{-1}$, in the group of patients who reached 100 mg $m^{-2}$ the mean dose intensity was 35.78 mg $m^{-2}$ $week^{-1}$, while for those who reached 110 and 120 mg $m^{-2}$ the mean dose intensity of EPI was 36.19 mg $m^{-2}$ $week^{-1}$ and 40.47 mg $m^{-2}$ $week^{-1}$ respectively. These differences in mean dose intensity were not statistically significant. No correlation was found between EPI dose levels and type or rate of objective response.

All patients experienced grade 3 alopecia. Gastrointestinal toxicity was rather mild. Grade 1–2 nausea/vomiting and stomatitis were recorded in 33 (54%) and 24 (39%) patients repectively, while grade 3 vomiting and stomatitis were recorded in 12 (20%) and five (8%) patients respectively. Diarrhoea was mild with only one case of grade 3. With regard to cardiotoxicity, one patient developed a myocardial infarction after seven cycles of a cumulative EPI dosage of 700 mg $m^{-2}$ and died 13 months after the beginning of chemotherapy of progressive cancer. In one other patient chemotherapy was stopped because of a 25% fall in LVEF after seven cycles at a cumulative EPI dosage of 760 mg $m^{-2}$. In a further three patients a 10% reduction in LVEF was noted but it did not fall below the normal limits: none of these patients developed any clinical sign of heart failure.

### *Objective responses and survival*

Overall, 61 out of 64 enrolled patients were evaluable for response. Three patients were lost to follow-up after the first cycle of chemotherapy before response evaluation. These patients were considered as treatment failures and thus response rates were calculated according to an intent to treat analysis considering all enrolled patients ($n = 64$).

Among the 57 patients with metastatic disease there were 14 CR (25%) and 27 PR (47%) with an overall response rate (ORR) of 72% (95% CL 66–78%), while nine patients (16%) had stable disease, and four (7%) progressed (three lost to follow-up). The median duration of CR and PR was 10 months and 13 months respectively. On the other hand, all seven patients with inoperable LABC achieved a PR and underwent radical mastectomy. All but one of these patients are alive and four are still disease free 18, 21, 31 and 32 months after surgery.

Analysis of response according to the site of metastases is shown in Table III. Soft tissue tumoral deposits (breast, skin and lymph nodes) as well as pleural effusions showed the highest response rates. No differences were seen between lung, pleural and liver lesions, while all pleural effusions

**Figure 1** WBC and ANC plotted against number of chemotherapeutic cycles. ●, median WBC; ■, median ANC.

**Figure 2** Platelet and haemoglobin values plotted against number of cycles. ●, haemoglobin values; ■, platelet values.

showed an objective response. Bone metastases responded in 65% of cases, but response rate at soft tissue metastases was higher than that recorded at bone lesions (87% *vs* 65%, $P<0.05$). Table IV shows analysis of response rates according to the dominant site of disease. As expected, patients with inoperable LABC showed the highest response rate, but not complete responses. Response rates did not vary significantly between the other groups. There was a difference in response rates between MBC patients with 1-2 sites of disease (81% ORR) and those with 3-4 sites (57% ORR), but the difference did not reach statistical significance. Among the 57 MBC patients who had previous adjuvant chemotherapy, 14 had a major response (CR + PR) for an overall response rate (61%); among the 34 patients who had not had adjuvant chemotherapy, 27 had a major response (79%). This difference was not statistically significant.

Among patients with MBC in whom sites of progressive disease were recorded, 43% relapsed in their on-study organ site of disease, 40% in previously disease-free organs and 17% in both sites. Progression of disease occurred in single or in multiple sites in 31 (65%) and 17 (35%) patients respectively, with a higher incidence of bone metastases (42%) followed by metastatic disease to the brain (19%).

After a median follow-up of 18 months (range 7-33 months) projected median survival of MBC patients was 20+ months. Among responding patients (CR + PR) there was a statistically significant difference in survival in favour of patients with bone-dominant site of disease as compared with those with visceral and soft tissue sites ($P=0.03$).

### Discussion

In the last 15 years the results of systemic treatment for advanced breast cancer have reached a plateau. Several efforts have been made to improve clinical results by increasing the dose intensity of chemotherapeutic treatments (Henderson *et al.*, 1988; Tannock *et al.*, 1988; Hortobagyi, 1990; Marschner *et al.*, 1994). In fact, haematopoietic growth factors have been employed as rescue from myelosuppression with the aims of increasing the dose of antineoplastic drugs and also shortening the inter-cycle interval (Bronchud *et al.*, 1989; O'Shaughnessy *et al.*, 1990; Piccart, 1990; Ten Bokkel Huinink and Clavel, 1990; Habeshaw *et al.*, 1991; Hoekman *et al.*, 1991; Gebbia *et al.*, 1994).

The aims of this study were to evaluate the possibility of increasing EPI dosage over 90 mg $m^{-2}$ $cycle^{-1}$, to identify the dose-limiting toxicity of EPI in combination with CTX and 5-FU/*l*-FA, employing a fixed dose of G-CSF and to test the clinical activity of such a regimen in advanced breast cancer. In this series of 61 patients the use of subcutaneous G-CSF allowed us to increase EPI by 10 mg $m^{-2}$ $cycle^{-1}$ above the starting dose level of 90 mg $m^{-2}$ $cycle^{-1}$ in 53 patients (87%), by 20 mg $m^{-2}$ in 31 patients (51%) and by 30 mg $m^{-2}$ in 18 cases (30%). An increase in frequency of administration was not possible in all patients in most instances because of myelotoxicity and mucositis. However, shortening of inter-cycle interval was possible in 20 patients but only for a few cycles because of cumulative toxicity. These data confirm, although only partially, the data reported by other authors, who were able to reduce the inter-cycle interval in a series of patients with MBC treated with high dose EPI + CTX (Piccart, 1990). It should be stressed that the chemotherapy regimen employed in the latter study was different from that given in our study.

In most patients the dose-limiting toxicity of high-dose EPI in combination with CTX and 3 day 5-FU/*l*-FA was myelotoxicity, even though three patients did not complete dose escalation because of mucosal toxicity. Analysis of haematological parameters showed a progressive decrease in median haemoglobin values, which was statistically correlated to the number of chemotherapeutic cycles. In fact, a median 2.7 g% loss of haemoglobin was recorded between basal and off-therapy values. A similar, but less marked, progressive increase in the severity of thrombocytopenia at nadir was also noted as the number of cycles increased. Moreover, although G-CSF rescue was possible in all cycles since differences in nadir WBC and ANC levels were not statistically significant, however G-CSF-induced spikes in ANC recorded before any cycle were progressively less marked as the number of chemotherapeutic cycles increased. These data suggest that sequential cycles of intensive chemotherapy with G-CSF marrow rescue may result in a cumulative myelosuppression which was evident as an unexpected high rate of significative anaemia, and in thrombocytopenia and leucopenia of increasing severity. This observation has been reported also by other authors (Hoekman *et al.*, 1991) employing granulocyte-macrophage colony-stimulating factor (GM-CSF) as rescue from myelosuppression induced by high-dose doxorubicin plus cyclophosphamide.

Although patients treated with EPI 120 mg $m^{-2}$ achieved a better dose intensity than patients treated at lower dose levels, these differences in dose intensity were not statistically significant and no correlation was found between EPI dose or dose intensity levels and type or rate of objective responses.

The ORR achieved in this multicentre study was 72% (95% CL 66-78%) with a 25% complete response rate. The median durations of complete and partial responses were 10 and 13 months respectively. The above-reported data confirm that this combination is active against MBC, achieving a high overall response rate similar to that reported with similar regimens (Hortobagyi, 1990; O'Shaughnessy *et al.*, 1990). Comparison of these results with those achieved by authors employing the same regimen with a lower dose of EPI, shows that higher doses of EPI may induce a 15% increase in ORR but a doubling in complete response rate (Palmeri *et al.*, 1991). However, it should be stressed that whether higher doses of EPI are associated with an increase in both rate and duration of objective response is beyond the aims of this study, and that this issue can be settled only in prospective randomised trials.

In conclusion, the DLT of EPI in combination with CTX and 3 day 5-FU/*l*-FA is represented by myelosuppression. These results further confirm that the use of G-CSF, given by a subcutaneous route, is effective in permitting an increased EPI dosage in such a combination regimen above the conventional dose, up to 120 mg $m^{-2}$ $cycle^{-1}$ in a significant percentage of patients. Intensive chemotherapy with G-CSF support may however sometimes result in very severe cumulative toxicity, which in the present study was represented by progressive anaemia and a trend towards

**Table III** Objective response according to single site of disease

| *Site* | *No. of patients* | *CR* | *PR* | *SD* | *PD* | *CR + PR (%)* |
|---|---|---|---|---|---|---|
| Viscera | | | | | | |
| Lung | 13 | 5 | 5 | 3 | – | 10 (77) |
| Liver | 16 | 6 | 6 | 2 | 2 | 12 (75) |
| Pleura | 4 | 3 | – | 1 | – | 3 (75) |
| Pleural Effusion | 6 | 4 | 2 | – | – | 6 (100) |
| Bone | 23 | 2 | 13 | 8 | – | 15 (65) |
| Soft tissue | | | | | | |
| Breast | 22 | 5 | 15 | 1 | 1 | 20 (91) |
| Lymph nodes | 40 | 20 | 13 | 5 | 2 | 33 (82) |
| Skin | 5 | 4 | 1 | – | – | 5 (100) |

**Table IV** Response according to dominant site of disease

| *Site* | *No. of patients* | *CR* | *PR* | *SD* | *PD* | *CR + PR (%)* |
|---|---|---|---|---|---|---|
| Viscera | 32 | 11 | 13 | 5 | 3 | 24 (75) |
| Bone | 14 | – | 11 | 3 | – | 11 (79) |
| Soft tissue | 8 | 3 | 3 | 1 | 1 | 6 (75) |
| LABC | 7 | – | 7 | – | – | 7 (100) |

increasing thrombocytopenia. We feel that EPI 110 mg $m^{-2}$ with G-CSF support can be employed in combination with CTX and 5-FU/*l*-FA on an outpatient basis, but with a close follow-up. The combination of CTX, 5-FU/*l*-FA and high-dose EPI with G-CSF support is highly effective against advanced breast cancer. However, this regimen cannot be recommended as routine standard therapy for metastatic breast cancer due to its significant toxicity and high cost, unless very aggressive and intensive chemotherapy is necessary.

### References

ALLEGRA C. (1991). Biochemical modulation: a modality that has come of therapeutic age. *J. Clin. Oncol.*, **9**, 1723-1726.

BONADONNA G, GIANNI L, SANTORO A, BONFANTE V, BIDOLI P, CASALI P, DEMICHELI R AND VALAGUSSA P. (1993). Drugs ten years later: epirubicin. *Ann. Oncol.*, **4**, 359-369.

BRONCHUD MH, HOWELL A, CROWTHER D, HOPWOOD P, SOUZA L AND DEXTER TM. (1989). The use of granulocyte colony stimulating factor to increase the intensity of treatment with doxorubicin in patients with advanced breast and ovarian cancer. *Br. J. Cancer*, **60**, 121-125.

CORTES EP, HOLLAND JF AND GLIDEWELL O. (1978). Amputation and adriamycin in primary osteosarcoma: a five year report. *Cancer Treat. Rep.*, **62**, 271-277.

FERGUSON JE, DODWELL DJ, SEYMOUR AM, RICHARD MA AND HOWELL A. (1993). High-dose intensive chemotherapy with doxorubicin and cyclophosphamide for the treatment of advanced breast cancer. *Br. J. Cancer*, **67**, 825-829.

FREI E AND CANNELLOS GP. (1980). Dose: a critical factor in cancer chemotherapy. *Am. J. Med.*, **69**, 585-594.

FRENCH EPIRUBICIN STUDY GROUP. (1988). A prospective randomized phase III trial comparing combination chemotherapy with cyclophosphamide, fluorouracil, and either doxorubicin or epirubicin. *J. Clin. Oncol.*, **6**, 679-688.

FRENCH EPIRUBICIN STUDY GROUP. (1991). A prospective randomized trial comparing epirubicin monochemotherapy to two fluorouracil, cyclophosphamide, and epirubicin regimens differing in epirubicin dose in advanced breast cancer patients. *J. Clin. Oncol.*, **9**, 305-312.

GEBBIA V, TESTA A, VALENZA R, CIPOLLA C, AMATO C, LATTERI M, GEBBIA N AND FLORENA M. (1992). Levofolinic acid and 5-fluorouracil plus high dose epidoxorubicin as first line treatment for metastatic breast carcinoma. *Anticancer Res.*, **13**, 141-144.

GEBBIA V, TESTA A, VALENZA R, BORSELLINO N, CIPOLLA C, CANNATA G, CURTO G, LATTERI MA, FLORENA M AND GEBBIA N. (1993). A prospective evaluation of the activity of human granulocyte colony stimulating factor on the prevention of chemotherapy-related neutropenia in patients with advanced carcinoma. *J. Chemother.*, **5**, 186-190.

GEBBIA V, VALENZA R, TESTA A, CANNATA G, VERDERAME F AND GEBBIA N. (1994). Escalating doses of mitoxantrone with granulocyte colony stimulating factor (G-CSF) rescue plus 5-fluorouracil and high dose levofolinic acid in metastatic breast cancer. *Eur. J. Cancer*, **11**, 1734-1736.

HABESHAW T, PAUL J, JONES R, STALLARD S, STEWARD M, KAYE SB, SOUKOUP M, SYMONDS RP, REED NS AND RANKIN EM. (1991). Epirubicin at two dose levels with prednisolone as treatment for advanced breast cancer: the results of a randomized trial. *J. Clin. Oncol.*, **9**, 295-304.

HENDERSON IC, HAYES DF, COME S AND GELMAN R. (1987). New agents and new medical treatments for advanced breast cancer. *Semin. Oncol.*, **14**, 34-36.

HENDERSON IC, HAYES DF AND GELMAN R. (1988). Dose-response in the treatment of breast cancer: a critical review. *J. Clin. Oncol.*, **6**, 1501-1515.

HOEKMAN K, WAGSTAFF J, VAN GROENINGEN CJ, VERMOKEN JB, BOVEN E AND PINEDO HM. (1991). Effects of recombinant human granuclocyte-macrophage colony-stimulating factor on myelosuppression induced by multiple cycles of high-dose chemotherapy in patients with advanced breast cancer. *J. Natl Cancer Inst.*, **83**, 1546-1553.

HOOGSTRATEN B. (1975). Adriamycin (NSC-123127) in the treatment of advanced breast cancer: studies by the Southwest Oncology Group. *Cancer Chemother. Rep.*, **6**, 329-339.

HORTOBAGYI GN. (1990). The importance of dose-response in cytotoxic therapy for breast cancer. In *Advances in Breast Cancer Treatment Therapeutic Strategies in Oncology*, Henderson IC and Broden EC (eds) pp. 47-69. Medescript: London.

HRYNIUK WM. (1987). Average relative dose intensity and the impact on design of clinical trials. *Semin. Oncol.*, **14**, 65-74.

ITALIAN MULTICENTRE BREAST STUDY IN EPIRUBICIN. (1988). Phase III randomized study of fluorouracil, epirubicin and cyclophosphamide versus fluorouracil, doxorubicin and cyclophosphamide in advanced breast cancer: an Italian Multicentre Trial. *J. Clin. Oncol.*, **6**, 976-982.

LOPRINZI CL. (1989). 5-Fluorouracil with leucovorin in breast cancer. *Cancer*, **63**, 1045-1047.

MARSCHNER N, KREIENBERG R, SOUCHON R, RATH U, EGGELING B, VOIGHTMAN R, RUFFERT K, SCHUTTE M, AMMON A, KESZTYUS T, KAPLAN E AND NAGEL G. (1994). Evaluation of the importance and relevance of dose intensity using epirubicin and cyclophosphamide in metastatic breast cancer: interim analysis of a prospective randomized trial. *Semin. Oncol.*, **21**, 10-16.

MILLER AB, HOOGSTRATEN B, STAQUET M AND WINKLER A. (1981). Reporting results of cancer treatment. *Cancer*, **47**, 207-214.

O'SHAUGHNESSY J, DENICOFF AM, DORR A AND TAYLOR J. (1990). Combination chemotherapy with fluorouracil (F), leucovorin (L), adriamycin (A), and cytoxan (C) with granulocyte macrophage colony stimulating factor (GM-CSF) for patients with stage III and stage IV breast cancer. *Proc. Am. Soc. Clin. Oncol.*, **9**, 47.

PALMERI S, GEBBIA V, RUSSO A, GEBBIA N, MELI M AND RAUSA L. (1991). Folinic acid (FA) and 5-fluorouracil (5FU) plus cyclophosphamide (CTX) and epiadriamycin (EDXR) in the treatment of metastatic breast carcinoma (MBC). *Eur. J. Cancer*, **22**, 58.

PARNES HL, ABRAMS JS, TAIT N, MINFORD J, ALLEN SL, DUGGAN D AND AISNER J. (1991). Phase I-II study of cyclophosphamide, doxorubicin, fluorouracil, and leucovorin for the treatment of metastatic adenocarcinoma. *J. Natl Cancer Inst.*, **83**, 1017-1020.

PICCART MJ. (1990). A review of G-CSF in the treatment of breast cancer. In *Advances in the Chemotherapy of Malignant Disease: Current and Future Applications of G-CSF in Oncology*. XV Congress of the European Society of Medical Oncology; 1 December; Copenhagen. p. 10.

TANNOCK IF, BOYED NF, DE BOER G, ERLICHMAN C, FINE S, LAROQUE G, MAYERS C, PERRAULT D AND SUTHERLAND H. (1988). A randomized trial of two dose levels of cyclophosphamide, methotrexate, and fluorouracil chemotherapy for patients with metastatic breast cancer. *J. Clin. Oncol.*, **6**, 1377-1387.

TEN BOKKEL HUININK WW AND CLAVEL M. (1990). Mitoxantrone (M) and GM-CSF: a phase I study with an escalated dose of M in breast cancer. *Proc. Am. Soc. Clin. Oncol.*, **9**, 42.

RAZAK E, VALERIOTE F AND VIETTI T. (1972). Survival of hematopoietic and leukemia colony forming cells in vitro following administration of daunorubicin or adriamicin. *Cancer Res.*, **32**, 1496-1500.

YATES JW, GLIDEWELL O, WIERNIK P, JONES S AND DUTCHER J. (1982). Activity of daunorubicin vs adriamycin induction and monthly vs bimonthly maintenance in acute myelocytic leukemia. *Blood*, **60**, 454-462.

ZANIBONI A, SIMONCINI E, MARPICATI P, MONTINI E, ARCANGELI G, MERIGGI F AND MARINI G. (1989). Cyclophosphamide, epirubicin, high dose folinic acid and 5-fluorouracil(super-fec) as first line chemotherapy for advanced breast cancer. *Eur. J. Cancer Clin. Oncol.*, **24**, 1151-1155.


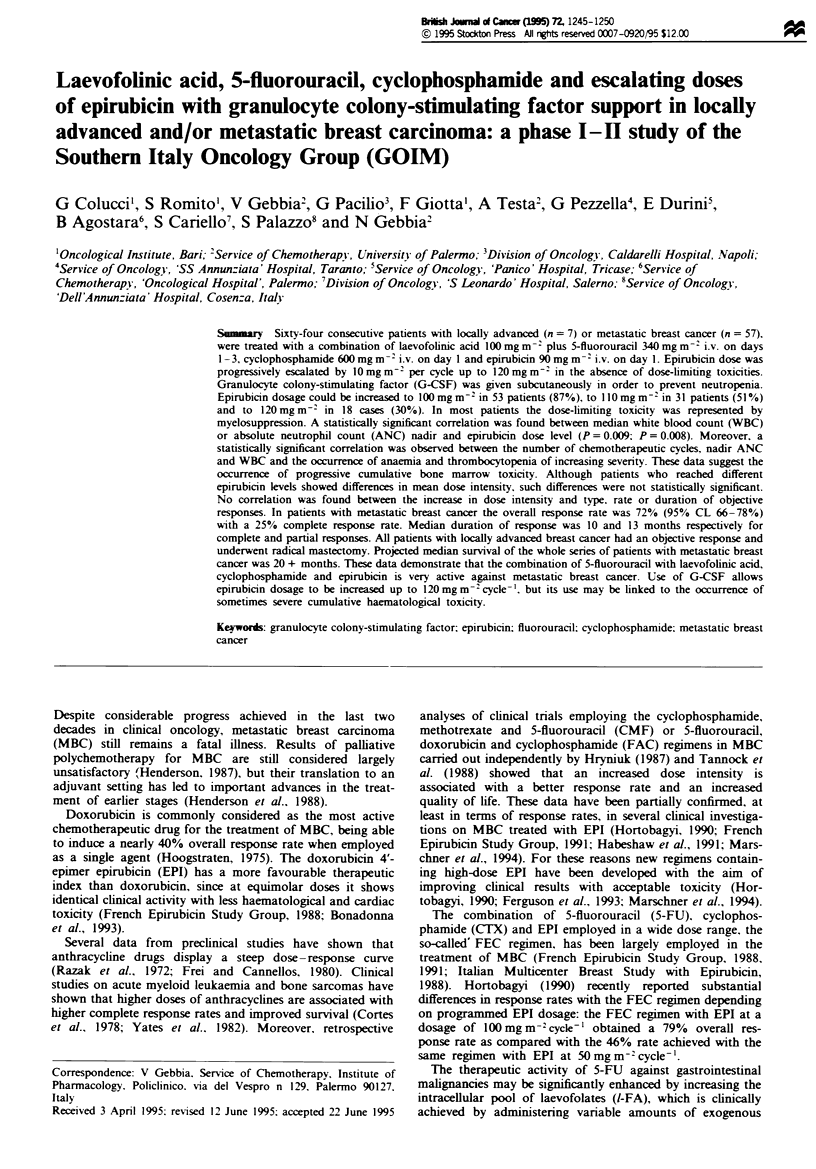

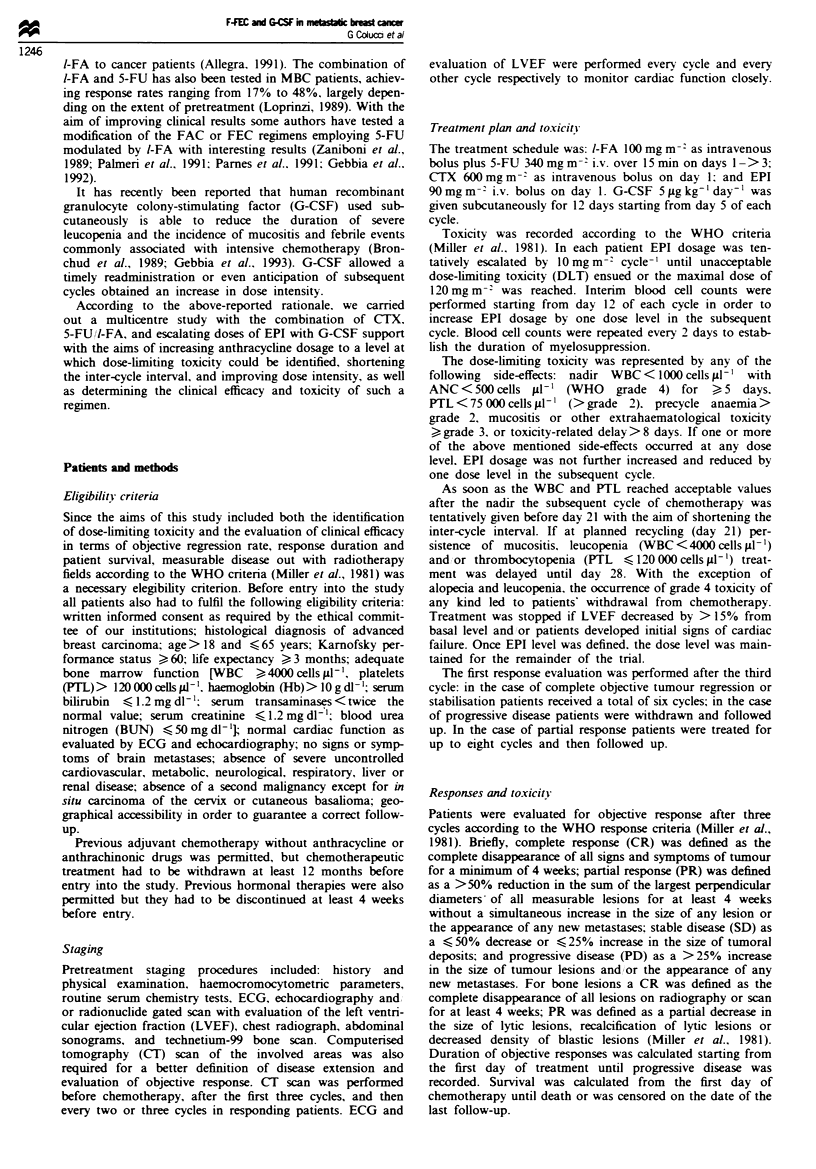

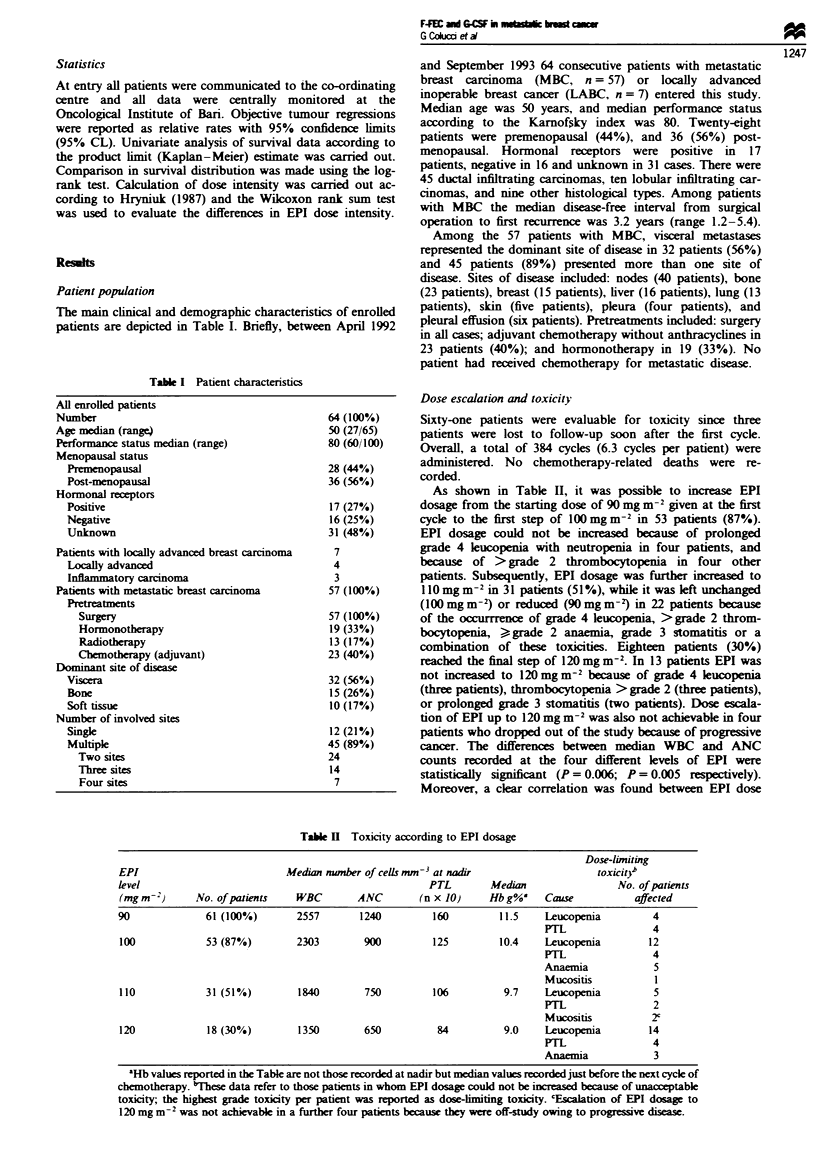

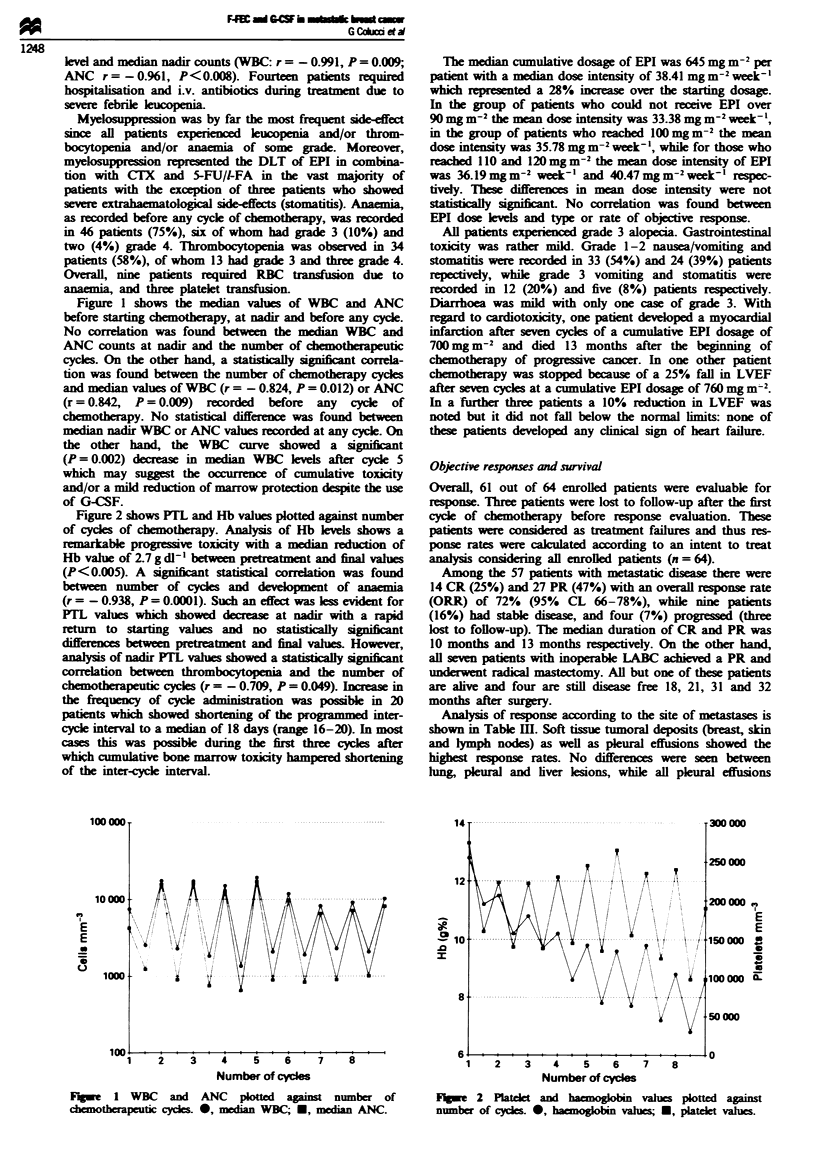

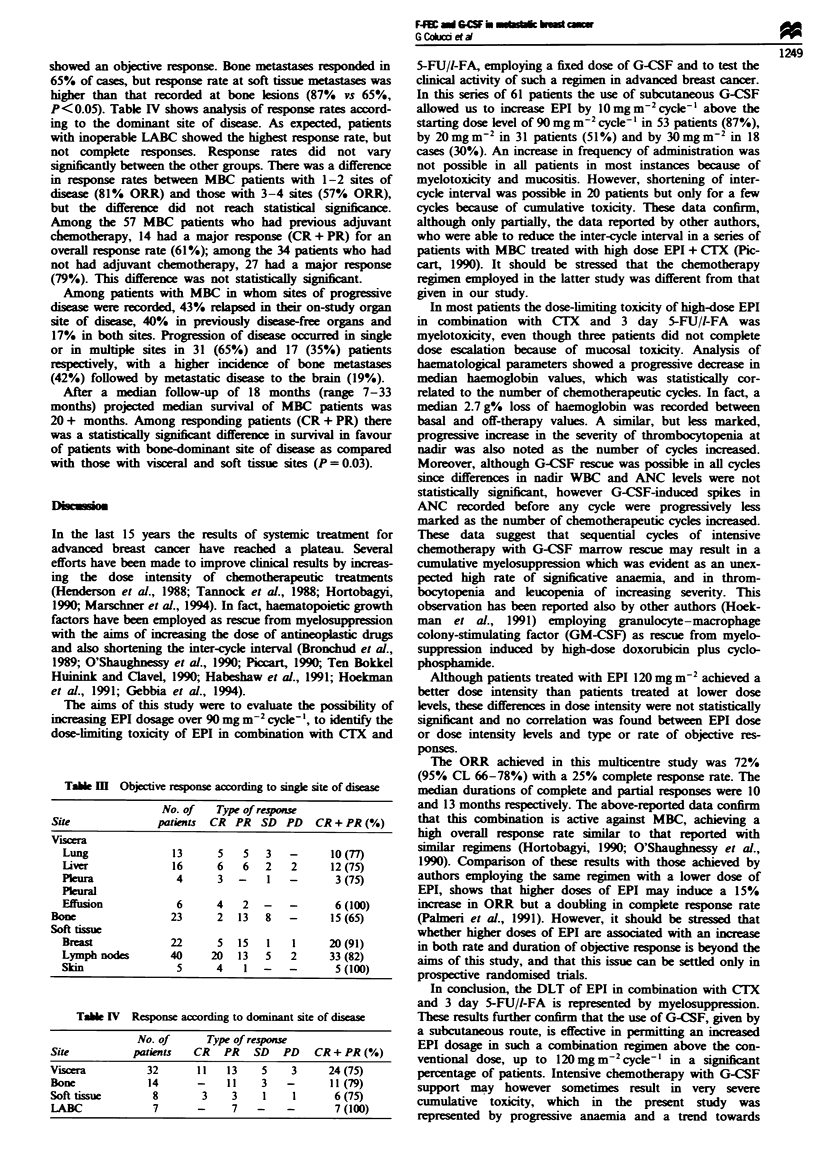

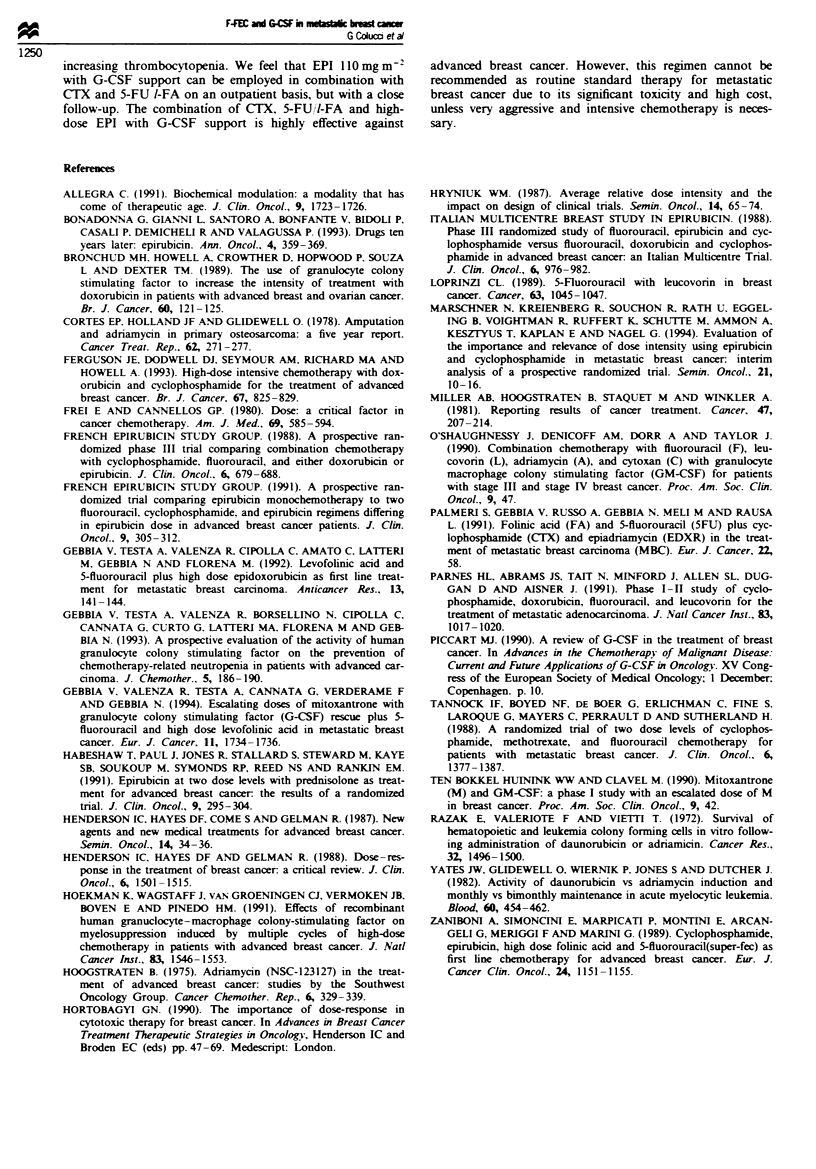


## References

[OCR_00843] Allegra C. J. (1991). Biochemical modulation: a modality that has come of therapeutic age.. J Clin Oncol.

[OCR_00850] Bonadonna G., Gianni L., Santoro A., Bonfante V., Bidoli P., Casali P., Demicheli R., Valagussa P. (1993). Drugs ten years later: epirubicin.. Ann Oncol.

[OCR_00852] Bronchud M. H., Howell A., Crowther D., Hopwood P., Souza L., Dexter T. M. (1989). The use of granulocyte colony-stimulating factor to increase the intensity of treatment with doxorubicin in patients with advanced breast and ovarian cancer.. Br J Cancer.

[OCR_00859] Cortes E. P., Holland J. F., Glidewell O. (1978). Amputation and adriamycin in primary osteosarcoma: a 5-year report.. Cancer Treat Rep.

[OCR_00866] Ferguson J. E., Dodwell D. J., Seymour A. M., Richards M. A., Howell A. (1993). High dose, dose-intensive chemotherapy with doxorubicin and cyclophosphamide for the treatment of advanced breast cancer.. Br J Cancer.

[OCR_00872] Frei E., Canellos G. P. (1980). Dose: a critical factor in cancer chemotherapy.. Am J Med.

[OCR_00894] Gebbia V., Testa A., Valenza R., Borsellino N., Cipolla C., Cannata G., Curto G., Latteri M., Florena M., Gebbia N. (1993). A prospective evaluation of the activity of human granulocyte-colony stimulating factor on the prevention of chemotherapy-related neutropenia in patients with advanced carcinoma.. J Chemother.

[OCR_00887] Gebbia V., Testa A., Valenza R., Cipolla C., Amato C., Latteri M., Gebbia N., Florena M. (1993). Levo folinic acid and 5-fluorouracil plus high dose epidoxorubicin as first line treatment for metastatic breast carcinoma.. Anticancer Res.

[OCR_00902] Gebbia V., Valenza R., Testa A., Cannata G., Verderame F., Gebbia N. (1994). Escalating doses of mitoxantrone with granulocyte colony-stimulating factor (G-CSF) rescue plus 5-fluorouracil and high-dose levofolinic acid in metastatic breast cancer.. Eur J Cancer.

[OCR_00912] Habeshaw T., Paul J., Jones R., Stallard S., Stewart M., Kaye S. B., Soukop M., Symonds R. P., Reed N. S., Rankin E. M. (1991). Epirubicin at two dose levels with prednisolone as treatment for advanced breast cancer: the results of a randomized trial.. J Clin Oncol.

[OCR_00918] Henderson I. C., Hayes D. F., Gelman R. (1988). Dose-response in the treatment of breast cancer: a critical review.. J Clin Oncol.

[OCR_00929] Hoekman K., Wagstaff J., van Groeningen C. J., Vermorken J. B., Boven E., Pinedo H. M. (1991). Effects of recombinant human granulocyte-macrophage colony-stimulating factor on myelosuppression induced by multiple cycles of high-dose chemotherapy in patients with advanced breast cancer.. J Natl Cancer Inst.

[OCR_00947] Hryniuk W. M. (1987). Average relative dose intensity and the impact on design of clinical trials.. Semin Oncol.

[OCR_00958] Loprinzi C. L. (1989). 5-Fluorouracil with leucovorin in breast cancer.. Cancer.

[OCR_00960] Marschner N., Kreienberg R., Souchon R., Räth U., Eggeling B., Voigtmann R., Ruffert K., Schütte M., Ammon A., Kesztyüs T. (1994). Evaluation of the importance and relevance of dose intensity using epirubicin and cyclophosphamide in metastatic breast cancer: interim analysis of a prospective randomized trial.. Semin Oncol.

[OCR_00971] Miller A. B., Hoogstraten B., Staquet M., Winkler A. (1981). Reporting results of cancer treatment.. Cancer.

[OCR_00989] Parnes H. L., Abrams J. S., Tait N., Minford J., Allen S. L., Duggan D., Aisner J. (1991). Phase I/II study of cyclophosphamide, doxorubicin, fluorouracil, and leucovorin for treatment of metastatic adenocarcinoma.. J Natl Cancer Inst.

[OCR_01018] Razek A., Valeriote F., Vietti T. (1972). Survival of hematopoietic and leukemic colony-forming cells in vivo following the administration of daunorubicin or adriamycin.. Cancer Res.

[OCR_01006] Tannock I. F., Boyd N. F., DeBoer G., Erlichman C., Fine S., Larocque G., Mayers C., Perrault D., Sutherland H. (1988). A randomized trial of two dose levels of cyclophosphamide, methotrexate, and fluorouracil chemotherapy for patients with metastatic breast cancer.. J Clin Oncol.

[OCR_01022] Yates J., Glidewell O., Wiernik P., Cooper M. R., Steinberg D., Dosik H., Levy R., Hoagland C., Henry P., Gottlieb A. (1982). Cytosine arabinoside with daunorubicin or adriamycin for therapy of acute myelocytic leukemia: a CALGB study.. Blood.

[OCR_01028] Zaniboni A., Simoncini E., Marpicati P., Montini E., Ferrari V., Ferragni A., Boari L., Marini G. (1989). Cyclophosphamide, epirubicin, high-dose folinic acid and 5-fluorouracil (super-FEC) as first-line chemotherapy for advanced breast cancer: preliminary results.. Eur J Cancer Clin Oncol.

